# Depurinized milk downregulates rat thymus MyD88/Akt/p38 function, NF-κB-mediated inflammation, caspase-1 activity but not the endonuclease pathway: *in vitro*/*in vivo* study

**DOI:** 10.1038/srep41971

**Published:** 2017-02-08

**Authors:** Gordana Kocic, Andrej Veljkovic, Hristina Kocic, Miodrag Colic, Dusan Mihajlovic, Katarina Tomovic, Svetlana Stojanovic, Andrija Smelcerovic

**Affiliations:** 1 Institute of Biochemistry, Faculty of Medicine, University of Nis, Bulevar Dr Zorana Djindjica 81, 18000 Nis, Serbia; 2 Medical Faculty, University Maribor, Magdalenski trg 5, Maribor 2000, Slovenia; 3 Medical Faculty of the Military Medical Academy, University of Defence in Belgrade, Crnotravska 17, 11 000 Belgrade, Serbia; 4 Department of Pharmacy, Faculty of Medicine, University of Nis, Bulevar Dr Zorana Djindjica 81, 18000 Nis, Serbia; 5 Department of Chemistry, Faculty of Medicine, University of Nis, Bulevar Dr Zorana Djindjica 81, 18000 Nis, Serbia

## Abstract

The aim of this study was the evaluation of 15 days dietary regimen of depurinized (DP) milk (obtained using our patented technological procedures) or 1.5% fat UHT milk instead of standard chow diet, on rat thymus and bone marrow MyD88/Akt/p38, NF-κB, caspase-1 and endonuclease pathways, in relation to peripheral blood cell composition. To determine whether the reduced mass of the thymus is a consequence of the direct effect of DP/UHT milk on apoptosis of thymocytes, *in vitro* Annexin-V-FITC/PI assay was performed. Significant decreases in the thymus wet weight, thymocyte MyD88, Akt-1/phospho-Akt-1 kinase, p38/phospho-p38, NF-κB, caspase-1 activity and CD4+/CD8+ antigen expression were obtained, especially in the DP milk group. The activity of thymocyte alkaline and acid DNase increased in the DP but not in the UHT milk group. The level of IL-6 significantly decreased in DP milk treated group, while the level of total TGF-β and IL-6 increased in UHT milk group. Significant differences in hematological parameters were obtained in commercial milk fed group. Observed results about prevention of experimental diabetes in DP pretreated groups may suggest that purine compounds, uric acid and other volatile toxic compounds of commercial milk may suppress oral tolerance, probably via IL-6 and TGF-β cytokine effects.

In recent years, the personalized nutrition concept has suggested that cow milk is a powerful epigenetic modulator of cell signaling, predominantly based on its endocrine components (insulin-like growth factor 1, IGF-1), essential amino acids, nucleotides and bioactive bovine exosomal microRNAs. It was documented that epigenetic cell signaling may have downstream targets, such as the nutrient-sensitive kinase rapamycin complex (mammalian target of rapamycin complex 1, mTORC1), which, upon phosphorylation, can exert an influence on the functional activity of cell transcription factors, DNA fragmentation and posttranscriptional RNA modification. This may modulate proliferation and intermediary metabolism, while in the thymic medulla, some exosome carriers may act as signals for immune communication^1^,^2^,^3^. Previous results, including ours, have shown that pasteurized milk and milk powder for infant formula contain only trace amounts of RNAs, but they contain different nucleotides, purine bases and uric acid. As components of human milk, nucleotides may contribute to the enhanced immunity of breast-fed infants^4^,^5^,^6^,^7^. Lactose, casein-degraded oligopeptides, nucleotides, uric acid and a number of chemical contaminants may determine susceptibility to systemic infection, immunoreactivity and the initiation and progression of chronic diseases, such as atherosclerosis, immune or neuro-cognitive diseases^6^,^7^,^8^,^9^,^10^,^11^. The mechanisms of their atherogenic effects were documented^11^,^12^. Regarding the lactose intolerance, it was documented that adult rats were not intolerant to levels of lactose less than or equal to 20% of daily diet intake. Continual dairy consumption, compared to a carbohydrate-based diet, may decrease systemic inflammation through a significant decrease in the expression of IL-1β and IL-6 and an improvement of hepatic steatosis index scores^13^,^14^. A recent clinical multicenter case-control study documented that adequate amounts of milk reduced the risk of malignant lymphoproliferative diseases and adult leukemia^15^.

Intestinal epithelium makes the first contact with ingested nutrients, recognizing pathogens or other non-pathogens with potentially immunogenic components. The primary function is to acquire immunological tolerance to normal dietary antigens. Up to now, some therapeutic approaches were enrolled, by using different protocols, to induce food tolerance for cow milk in children affected with cow milk protein allergy (CMPA). The protocols which introduced continuous administration of incremental doses of cow milk were documented in specific oral tolerance induction (SOTI) studies. Regarding the obtained results, proposed treatment may be promising in developing food tolerance, but specific protocol design should avoid possible risk and should be created in respect to individual clinical reaction^16^,^17^. The nutrigenomic and other stress-induced mechanisms that may regulate the thymus’s metabolic activity, followed by the depression of the thymus-dependent immune response, may be different. Despite its natural involution, it was documented that the adult thymus may substantially contribute to the peripheral immune response, which makes it a potential target for therapeutic interventions^18^,^19^,^20^. The depression of the thymus-dependent immune response is associated with the loss of immature cortical thymocytes, the volume of the thymic epithelium and a decreased number of helper to cytotoxic T lymphocytes (CD4^+^/CD8^+^ cells)^18^,^19^.

MyD88 (myeloid differentiation primary response gene 88) represents one adaptor protein that links the Toll-like receptor (TLR) family and interleukin-1 receptor (IL-1R) to the downstream activation of the transcription factor NF-κB, mitogen-activated protein kinases (p38 MAP kinase) and Akt kinase pathways. Stimulation of TLRs can bridge innate and adaptive immunity and can promote the synthesis of pro-inflammatory cytokines^21^,^22^,^23^,^24^,^25^,^26^. The presence of MyD88 in intestinal epithelial cells may serve as a sensor that modulates host metabolism according to specific food challenges. As a switcher, it may be a possible therapeutic target in obesity and metabolic disorders^27^,^28^.

In the cascade of signaling events permitting controlled and programmed cell death or apoptosis, the main executive events are terminal protein and DNA fragmentation. Apoptotic endonucleases are a family of DNases (acid and alkaline types) that represent the key enzymes in terminal DNA fragmentation^29^,^30^. Caspase-1 is known as Interleukin-1β converting enzyme (ICE) because it is capable of converting the precursors of the inflammatory cytokines IL-1β and IL-18 to active forms. The known substrate of caspase-1 was documented to be Bcl-xL as well. Thus, in addition to its apoptotic influence, caspase-1 may trigger inflammatory and autoimmune reactions^31^,^32^,^33^.

To determine the functional significance of purines and uric acid, we developed a technological procedure and filter device system to obtain milk almost free from purine compounds (depurinized-DP milk), but the mass spectrometry analyses documented that obtained milk is also free from more than 30 toxic volatile compounds^34^,^35^,^36^,^37^. In our previous reports, we documented the beneficial effect of DP milk on liver signaling related to the regeneration and improvement of cardiac markers during experimentally induced hyperuricemia^6^,^7^,^38^. Currently, it is unknown whether the MyD88/Akt/p38 signaling cascade or NF-κB-dependent inflammatory response may be involved in mediating any effect of depurinized milk on the functional activity of the thymus or bone marrow. The aim of our current experimental study was to examine the effect of a dietary milk regimen (DP or commercial 1.5% fat UHT milk) on rat thymic and bone marrow MyD88, Akt-1 kinase/phospho-Akt-1 kinase, p38/phospho-p38, NF-κB, endonuclease (acid and alkaline DNase) and caspase-1 activity in relation to the peripheral blood cell count and composition and plasma cytokine (IL-6 and total TGF-β) level. To test whether specific milk derivatives may directly alter thymocyte structure and cell membrane composition, phosphatidylserine (PS) externalization on cellular membranes was estimated in isolated rat thymocyte cultures. In order to explore if obtained oral tolerance may suppress development of diabetes, streptozotocin diabetes was employed as a model with different milk regimens.

## Results

### MyD88, Akt-1 kinase/phospho-Akt-1 kinase, p38/phospho-p38, NF-κB, CD4+/CD8+, DNase and caspase-1 determination in rat thymocytes

**Figure 1:**
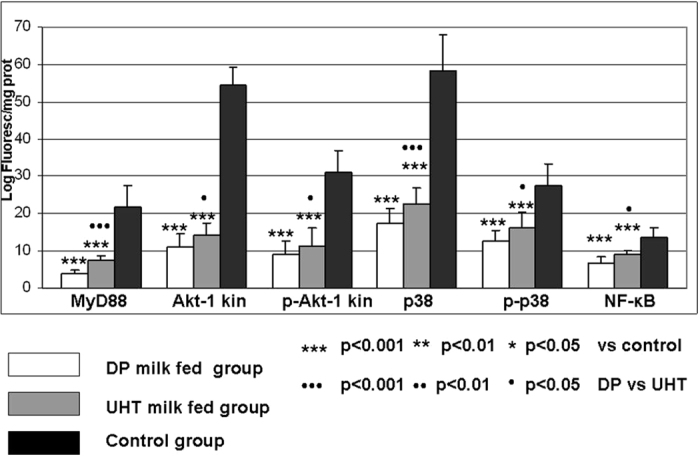
Thymocyte levels of MyD88, Akt-1 kinase, phospho-Akt-1 kinase, p38, phospho-p38 and NF-κB in rats fed different milk diets. Adult (eight weeks old) female Wistar rats were allocated into three dietary groups (8 rats/group). The first group received depurinized milk (DP milk) instead of standard laboratory chow for 15 days; the second group received only commercial UHT 1.5% cow’s milk instead of standard laboratory chow for 15 days; and the control group received standard laboratory chow. The indirect immunofluorescence assay was performed for measurement of the quantitative expression of parameters (logarithm of fluorescence per quantity of cellular proteins).

**Figure 2:**
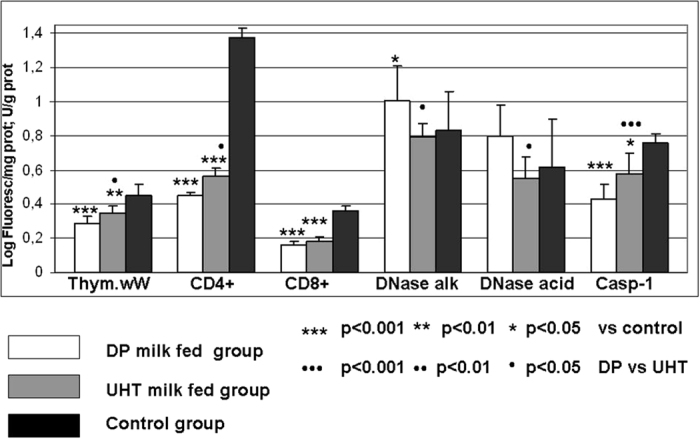
Thymocyte wet weight (TwW), CD4+ and CD8+ expression, alkaline DNase, acid DNase and caspase-1 activity in rats fed different milk diets. Adult (eight weeks old) female Wistar rats were allocated into three dietary groups (8 rats/group). The first group received depurinized milk (DP milk) instead of standard laboratory chow for 15 days; the second group received only commercial UHT 1.5% cow’s milk instead of standard laboratory chow for 15 days; and the control group received standard laboratory chow. The activity of acid and alkaline DNase was measured spectrophotometrically based on acid-soluble nucleotide determination after precipitation. Enzyme activity was expressed as U/g protein using corresponding standard commercial DNase I and DNase II. The indirect immunofluorescence assay was performed for measurement of the quantitative expression of CD4+, CD8+ (expressed as the logarithm of fluorescence per quantity of cellular proteins). Caspase-1 was determined using a caspase assay kit from RD Diagnostics and expressed as U/g protein.

The results of the quantitative determination of MyD88, Akt kinase/phospho-Akt kinase, p38/phospho-p38 and NF-κB in rat thymocytes are shown in Fig. 1. The thymus wet weight (wW) and thymocyte CD4+ and CD8+ expression, alkaline DNase, acid DNase and caspase-1 activity are shown in Fig. 2. DP milk and UHT milk led to significant decreases in thymocyte MyD88, Akt-1 kinase/phospho-Akt-1 kinase, p38/phospho-p38, NF-κB, CD4+ and CD8+ antigen expression; the CD4+ lineage was more affected than the CD8+; and the effect was more pronounced for the DP milk group (Figs 1 and 2). Both milk diets, especially DP, led to thymus involution and a substantial decline in wet weight (Fig. 2). Caspase-1 activity exhibited the lowest value in the DP milk group as well (Fig. 2). The activity of thymocyte alkaline and acid DNase was increased in the DP milk group but not in the commercial UHT milk group.

### MyD88, Akt-1 kinase/phospho-Akt-1 kinase, p38/phospho-p38, NF-κB and DNase determination in rat bone marrow

**Figure 3:**
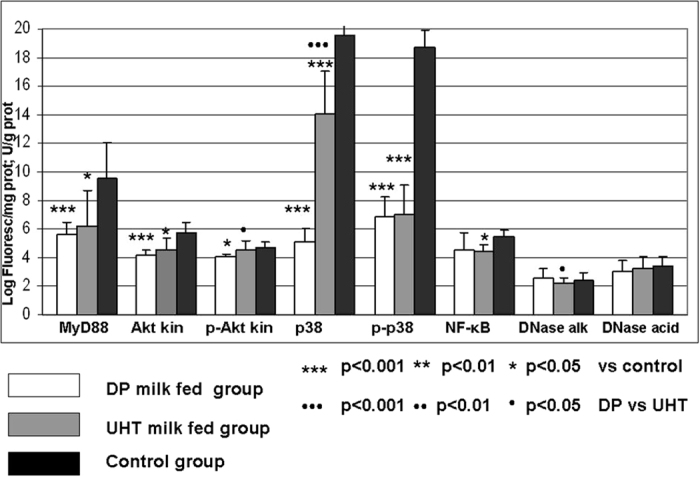
Bone marrow MyD88, Akt-1 kinase, phospho-Akt-1 kinase, p38, phospho-p38, NF-κB, alkaline DNase and acid DNase in rats fed different milk diets. Adult (eight weeks old) female Wistar rats were allocated into three dietary groups (8 rats/group). The first group received depurinized milk (DP milk) instead of standard laboratory chow for 15 days; the second group received only commercial UHT 1.5% cow’s milk instead of standard laboratory chow for 15 days; and the control group received standard laboratory chow. The indirect immunofluorescence assay was performed to measure the quantitative expression of MyD88, Akt-1 kinase, phospho-Akt-1 kinase, p38, phospho-p38 and NF-κB (expressed as the logarithm of fluorescence per quantity of cellular proteins). The activity of acid and alkaline DNase was measured spectrophotometrically based on acid-soluble nucleotide determination after precipitation. Enzyme activity was expressed as U/g protein using corresponding standard commercial DNase I and DNase II.

We next examined these parameters for bone marrow, and almost the same expression pattern was documented for MyD88, Akt-1 kinase/phospho-Akt-1 kinase, p38/phospho-p38, NF-κB, alkaline DNase and acid DNase (Fig. 3), with a significant decrease in the above-mentioned parameters, especially in the DP milk group.

### Cytokine analysis

**Figure 4:**
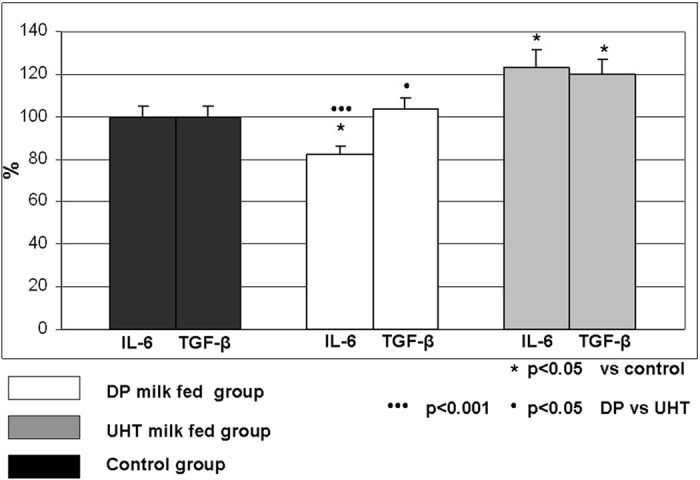
Plasma level of IL-6 and total TGF-β in rats fed different milk diets. Cytokines TGF-β and IL-6 were measured by using standard ELISA test for quantitative determination in plasma. The level was expressed as percent of control value.

Our experimental results documented significant decrease of IL-6 level, but not of total TGF-β in plasma of rats receiving DP milk, while the level of IL-6 and total TGF-β increased in rats feeding UHT 1.5% commercial cow milk (Fig. 4).

### *In vitro* apoptosis assay of isolated thymocytes

**Figure 5:**
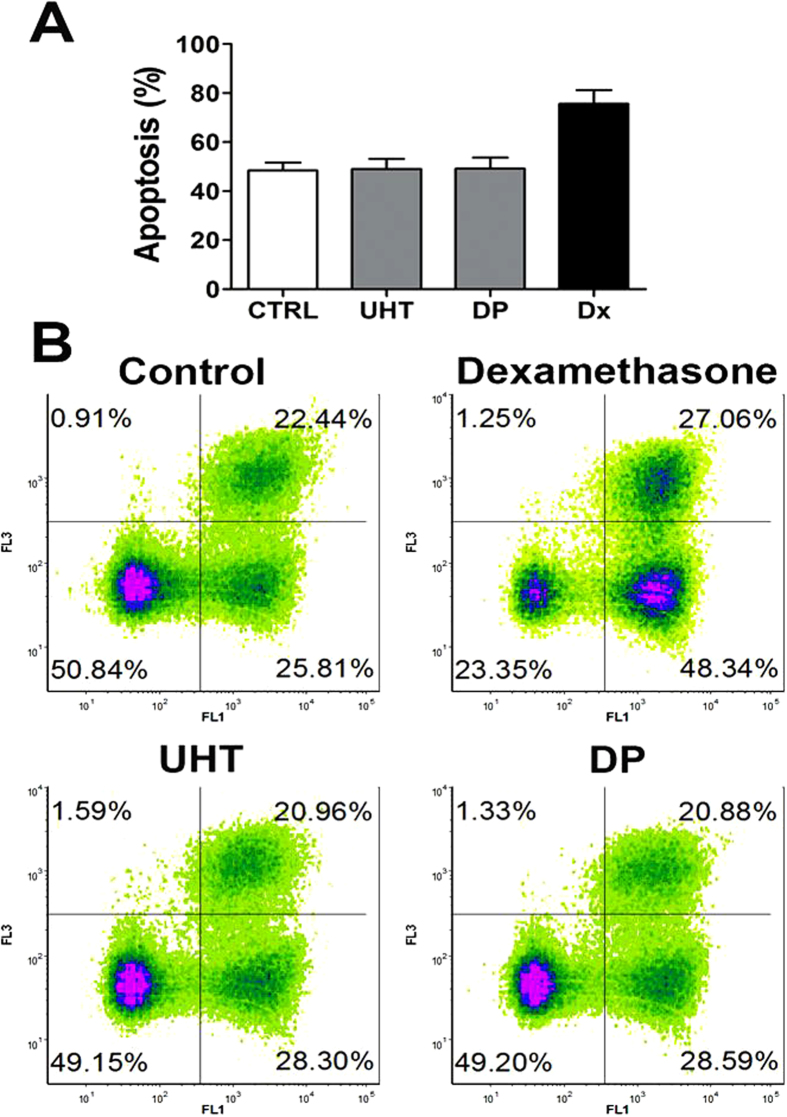
The effect of UHT milk and DP milk on the apoptosis of isolated rat thymocyte cultures. Thymocytes were cultivated with different concentrations of UHT milk, DP milk or in a control medium for 20 hours. Then, the cells were stained with Annexin-V-FITC/PI and analyzed by flow cytometry. Values are given as percentages of apoptotic cells (mean ± SD, n = 6) (**A**) or as the representative density plots (**B**).

To determine whether the observed effects of UHT and DP milk on reduced mass and cellularity of the thymus were a consequence of the direct effect of milk on the apoptosis of thymocytes, we established a classical thymocyte apoptosis assay *in vitro* using an Annexin-V-FITC/PI assay. The results presented in Fig. 5 show that neither UHT nor DP milk applied to the culture of isolated thymocytes at various concentrations (1:100) induced apoptosis, in contrast to the positive control (dexamethasone). Lower dilutions (1:50) yielded the same results (data not shown).

### Hematological parameters analysis

Furthermore, we analyzed the peripheral blood cell count and plasma protein characteristics. The significant differences in blood hematological parameters (erythrocytes, leucocytes and platelets) were obtained in groups fed commercial milk instead of commercial chow. The significant decrease in red blood cell count (RBC), significant decrease in hemoglobin level and significant increase in total white blood cell count (WBC) were not documented in the DP milk group (Table 1). Blood hematocrit was significantly lower in the commercial milk group, which may correlate with the decreased RBC number. Interestingly, the results from the DP milk group were very close to those of the control, despite the changes obtained in thymocytes and bone marrow. Regarding the profile of WBCs, a shift with a decline in the peripheral lymphocyte count and an increase in neutrophil count was documented in the commercial milk group. Platelet changes were significant for DP milk rats with a significant decrease in platelet count. This was followed by hypoproteinemia in plasma with a significant decrease in serum albumin (Table 1).

### Streptozotocin-induced diabetes in rats

**Figure 6:**
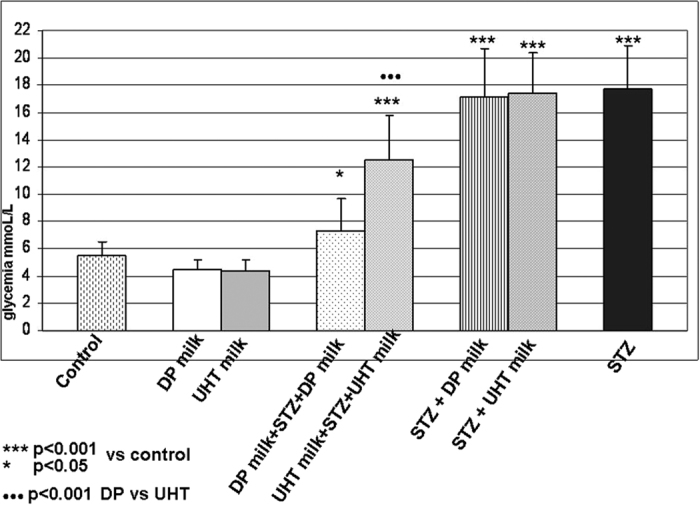
The effect of different milk regimen treatment on glycemia level in experimental streptozotocin (STZ) diabetes. The rats were allocated into eight experimental groups, the experiment lasted 14 days. Streptozotocin diabetes was induced by a single intraperitoneal injection of Streptozotocin in a dose of 62 mg/kg body weight. The following eight groups were allocated: i) control group receiving standard diet (STD); ii) group fed DP milk for 14 days, instead of STD; iii) group fed commercial UHT 1.5% cow milk instead of STD, for 14 days; iv) group fed DP milk for 7 days before STZ injection and continued for next 7 days after the STZ; v) group fed commercial UHT 1.5% cow milk for 7 days before STZ injection and continued for next 7 days after STZ; vi) group received simultaneously STZ injection and fed DP for next 7 days; vii) group received simultaneously STZ injection and fed commercial UHT 1.5% cow milk for next 7 days; viii) group received STZ injection and fed STD for next 7 days. No insulin was administered. The diabetic state was confirmed by the measuring of blood glucose levels.

Diabetes was induced in rats by a single intraperitoneal injection of streptozotocin. A group with streptozotocin diabetes fed with standard laboratory chow diet (STD) served as diabetic control, while control rats were on STD only. Two groups received DP milk and commercial UHT 1.5% cow milk as it was explained in a study protocol (Materials and methods). In a group DP milk pretreated after streptozotocin injection, observed hyperglycemia, as the sign of streptozotocin-induced diabetes was only slightly increased, compared to diabetic rats, while in a group pretreated with commercial UHT 1.5% cow milk, it was only moderately increased, compared to corresponding group of streptozotocin diabetes on STD. Corresponding groups which received streptozotocin injection and DP or commercial UHT 1.5% cow milk simultaneously for 7 days, did not show any difference compared to streptozotocin diabetes on STD (Fig. 6).

## Discussion

Our experimental study showed that compared to dietary milk regimens with commercial UHT cow milk, the newly patented DP milk diet led to significant thymus atrophy after feeding rats for 15 days, which was absolute (thymus wet weight) and relative (thymus wet weight/body weight). No significant changes were observed in relation to body weight after milk diet treatment (Table 1). The milk diets were equilibrated for daily protein intake with the protein content of standard laboratory chow given to control rats.

The increased incidence of a number of immune-mediated and chronic inflammatory diseases and states, such as obesity, insulin resistance and type 2 diabetes; autoimmune diseases, such as type 1 diabetes, rheumatoid arthritis, inflammatory bowel disease and psoriasis; and malignant lymphoproliferative diseases may highlight the need to adjust dietary recommendations according to specific needs. High levels of circulating oligonucleotides and serum uric acid were considered as independent risk factors and DAMPs (damage-associated molecular patterns) in the deterioration of all of the above-mentioned diseases or conditions and should be considered seriously^12^,^39^ according to the tissue-specific response.

MyD88 is one central adaptor protein of TLRs that is responsible for the downstream stimulation of many effectors of the innate and adaptive immune responses, including TNF receptor-associated factor 6 (TRAF6), phosphoinositide 3-kinase (PI3K)/Akt, mitogen-activated protein kinases (MAPKs) and NF-κB. The PI3K-Akt pathway is involved in the activation in CD8+ T cells and the co-stimulation of CD4+ T cells. The Akt pathway represents a key signal responsible for the activation of thymocyte survival, proliferation, differentiation, maturation and clonal selection. Among the three products of the mammalian *akt* gene family (Akt-1, Akt-2, Akt-3), Akt-1 and Akt-2 are documented in the thymus and are mainly responsible for early T cell development^40^,^41^,^42^. The Akt kinase is activated by the phosphorylation of its hydrophobic tail at the serine 473 position and the activation motif of threonine at the 308 position. Using a phospho-specific anti-Akt-1 antibody, we detected the quantity of phosphorylated Akt-1 kinase. The causal link between lymphocyte proliferation and Akt kinase was documented in transgenic mice expressing a constitutively active form of T lymphocyte Akt kinase^40^. The dysregulated cell proliferation and the appearance of neoplastic morphology were documented in the experimentally upregulated Akt pathway^42^,^43^. The MyD88 adaptor protein-stimulated pathway may induce the activation of NF-κB, a main inflammatory transcription factor. The phosphorylation of IκB kinase-related kinase (IKKβ) causes κB dissociation and the formation of the active NF-κB p65 subunit. This event can be subsequently followed by the nuclear translocation of NF-κB p65, which induces the transcription of the pro-inflammatory cytokines TNF and IL-6. We report here that a milk diet, especially DP type, may attenuate the immune inflammatory response mediated via MyD88/Akt, MAPKs p38 and NF-κB p65. Some dietary regimens, including protein malnutrition, may induce depression of the thymus-dependent immune response, followed by the decrease in T helper CD4+ cells and the decline in the CD4+/CD8+ ratio. The results of a recent clinical multicenter study documented that an adequate amount of milk may reduce the risk of adult leukemia^15^. The obtained clinical results may be explained in terms of a significantly reduced thymus size and reduced Akt-1 and phospho-Akt-1 kinase after a milk diet. In addition, DP milk significantly depressed the MyD88/Akt/p38 and NF-κB pathways in bone marrow more than commercial milk (Fig. 3). T cell systemic hyporesponsiveness, or T cell anergy, due to the adaptive oral tolerance, has been mostly concerned with the functional inactivity of CD4^+^ and CD8^+^ cells, followed by their suppressed proliferation via inhibition of MAP kinase pathway^44^. Further results have disclosed that oral tolerance was followed by the inactivation of the CD4^+^ cells only, what may maintain systemic immune quiescence^45^,^46^. High dose oral antigen suppression is known as bystander suppression^47^,^48^. Extramedullary hematopoiesis, the proliferation of hematopoietic cells outside of the bone marrow, may occur in specific conditions when bone marrow hematopoiesis is suppressed^49^. In our study, the milk regimens did not replace hematopoiesis in the spleen, as significant spleen atrophy was also documented (Table 1). Observed peripheral hypochromic microcytic anemia and a significant decrease in hemoglobin may be explained by the well-documented effect of milk in triggering anemia because of the relatively low iron content in cow’s milk and decreased iron bioavailability^49^,^50^. The group of rats fed DP milk may be protected because this milk is almost free from toxic volatile compounds that are capable of diminishing hematopoiesis.

The modulation of Akt kinase was documented to be cell- and tissue-specific, as the overexpression of Akt/phospho-Akt kinase and Erk/phospho-Erk kinase in liver tissue and the increase in liver regenerative potential were obtained in our previous report after feeding animals with DP and commercial milk^6^.

We further addressed whether the silencing of Akt/phospho-Akt kinase may affect the apoptosis pathway and endonuclease and caspase-1 activity (Fig. 2). DNases are members of the DNA-degrading pathway, catalyzing the terminal step of the hydrolytic cleavage of phosphodiester linkages of genomic DNA^29^. The optimum pH values of acid and alkaline DNase of the rat thymus are 5.3 and 8.5, respectively. Acid DNase may be active in the absence of added divalent cations, while alkaline DNase is active in the presence of Mg ions. The role of DNase γ in the apoptosis of the thymus was documented, but our study did not distinguish the specific activity of DNase γ to confirm its role in thymocyte atrophy. Previous results documented that alkaline DNase may be involved in T cell differentiation^30^,^51^. The obtained results (Fig. 2) documented increased alkaline and acid DNase activity only in the DP milk group of rats, which suggested that only DP milk accelerates T cell apoptosis, which may be important in lymphoproliferative states. Caspase-1, also known as the Interleukin 1β converting enzyme (ICE), is important in the inflammatory response because of its ability to form the active cytokines IL-1 β and IL-18 from their inactive precursors. Because of the mentioned catalytic activity, it was suggested to play a larger role in inflammation than in apoptosis. Active caspase-1 is derived from the activation of the caspase cascade, where the cleavage of the 45-kDa pro-caspase molecule occurs by caspase-11, generating two fragments (20 kDa and 10 kDa), both with independent catalytic sites. Caspase-1 may also influence thymic stromal lymphopoietin (TSLP) production, which induces the differentiation of naive CD4+ T cells, which are implicated in the pathogenesis of allergic diseases^32^,^33^,^52^. In this way, the obtained results regarding DP milk may provide the opportunity to treat autoimmune, allergic or chronic inflammatory diseases. By using oral tolerance model for possible suppression of experimental multiple sclerosis development in mice, it was documented that the reduction of Th1 response was followed by the reduced secretion of a number of pro-inflammatory cytokines, such as IL-1α, IL-6, IL-9, IL-12p70, MIP-1β, RANTES and Eotaxin^53^. From the other side, it was recently documented that the low doses of oral antigens may induce oral tolerance by induction of latent TGF-β (LAP TGF-β) by Th3 like cells, what was significantly suppressed by IL-6. In this way, a possible blocking of IL-6 receptor or IL-6 signaling may enhance oral tolerance^54^. As the main effector molecule capable of contributing to the dietary antigens-induced oral tolerance, was proposed to be the TGF-β^55^. But the role of TGF-β1 in oral tolerance induction was questioned by using a model of TGF-β1 null mice, where it was documented that high oral doses of ovalbumin exhibited highly significant suppression of different lymph node cells, as it intact mice^56^. Oral tolerance has also been induced in IL-4-deficient mice, not requiring active Th2 cells^46^. In trying to find a possible influence of IL-6/latent TGF-β axis, we measured these cytokines in plasma of experimental animals. Our experimental results documented significant decrease of IL-6 level, but not of total TGF-β in plasma of rats receiving DP milk, while the level of IL-6 and total TGF-β increased in rats feeding commercial milk (Fig. 4). Observed effects may be explained in terms of enhanced immunomodulating properties of nucleotides^57^ and uric acid, where the action of uric acid as an immune adjuvant was also documented^58^. Since induction of TGF-β in culture of intestinal epithelial cells was not observed after their exposure to different nucleotide monophosphates or their mixture^59^, it may suggest that their complex immune-mediating effects are not mediated by intestinal cells, but rather to the complex interaction with surrounding immune cells, bacteria and/or systemic immune reaction.

To test whether milk derivatives directly alter thymocyte structure and cell membrane composition, phosphatidylserine (PS) externalization on the cellular membrane was performed. PS externalization at the external membrane leaflet is a marker of the early phase of apoptosis. No specific effect of Annexin V binding to PS on the external thymocyte membrane was documented during the treatment of *in vitro* thymocyte cultures with DP or commercial milk (Fig. 5).

The concept of acquired immune tolerance to specific orally-administrated antigen, have already been proved as effective in preventing experimental diabetes^60^. In order to test if observed immune hyporesponsiveness may prevent or modulate development of experimental diabetes after milk regimens-induced oral tolerance, we have involved next experiment, where streptozotocin diabetes was induced in rats. The rats were allocated into eight experimental groups, the experiment lasted 14 days. DP milk or commercial UHT 1.5% cow milk were given 7 days before and continued 7 days after the streptozotocin injection and the observed effect on diabetes development was compared to the groups where the animals received simultaneously streptozotocin and DP or commercial milk in 7 days. A group with streptozotocin diabetes fed with STD served as diabetic control, while control rats were on STD only. Two groups received DP milk and commercial UHT 1.5% cow milk respectively. In a group DP milk pretreated, hyperglycemia, as the sign of streptozotocin-induced diabetes, was only slightly increased, while in a group pretreated with commercial UHT 1.5% cow milk, it was only moderately registered, compared to corresponding group of streptozotocin diabetes on STD. Corresponding groups which received simultaneously streptozotocin and DP or commercial UHT 1.5% cow milk in 7 days did not show any difference compared to streptozotocin diabetes on STD (Fig. 6). Overall, DP milk pretreatment induced dramatic streptozotocin diabetes attenuation, more than commercial UHT 1.5% cow milk pretreatment. Observed results have documented that a possible non-specific systemic immune suppression may be counteracted by immunomodulatory activity of presenting nucleotides and uric acid, what would be further elucidated in our next study.

In conclusion, experimental rodent dietary design may provide a way to translate obtained effects in human nutrition research, through appropriate milk composition and its individual dosing. Our results have shown that DP milk may induce thymic downregulation of MyD88/Akt-1/p38 kinase proliferative pathway, which may suppress inflammation via the NF-κB and caspase-1 pathway and might be suitable therapeutic approach in the magnitude of human disease, such as hyperuricemic states, metabolic, immunoinflammatory, allergic and lymphoproliferative diseases. Observed results about prevention of experimental streptozotocin-diabetes only in DP pretreated groups, may suggest that purine compounds, uric acid and some volatile toxic compounds may suppress oral tolerance, probably via IL-6 and TGF-β cytokine interaction.

## Material and Methods

### Depurinized milk (DP milk) production

Commercial UHT cow milk with 1.5% fat content was purchased from Nis Dairy Industry. To obtain DP milk we used our patented technological procedures and the filter device system^34^,^35^,^36^,^37^. The nutritional characteristics of DP milk (protein, lactose and lipids) were determined, and they did not differ significantly from the untreated commercial milk. Further mass spectrometry analyses documented that DP milk is almost completely free from uric acid and from more than 30 potentially toxic volatile compounds, such as three types of sulfate derivatives (dimethylsulfoxide, dimethylsulfone, dimethylsulfide), aldehydes (nonanal), nine ketones (from propanones to heptanone or *n*-decanone), four alcohols (from ethanol to 1-hexanol), esters of hexanoic and octanoic acid, five types of acids and four types of phthalates (di-*n*-butyl phthalate, benzyl butyl phthalate, bis(2-ethylhexyl) phthalate and di-*n*-octyl phthalate^6^.

### Ethics Statement

All experimental procedures were performed according to the Ethical Committee guidelines and rules for the protection of the welfare of experimental animals adopted by the Medical Faculty University Nis, according to the Animal Welfare Rules Republic of Serbia. The approval for the proposed animal experiment was obtained from the Animal Welfare Committee N° 21-4545-2/9 (University Nis Medical Faculty) after submitting the required form for approval with the detailed experimental protocol.

### Animals

Adult female Wistar rats, weighing 200 ± 10 g and aged eight weeks, were obtained from the breeding colony of the Biomedical Research Centre University Nis Medical Faculty. The animals were fed with standard commercial food chow and water *ad libitum,* housed in a temperature-controlled room at 25 ± 3 °C and relative humidity of 50 ± 5% on a 12 hours light/12 hours dark cycle. A standard laboratory chow diet (STD) was given at a daily dose of 20 g per rat (equivalent to 4 g of protein daily). The rats were allocated into three dietary groups (8 rats/group). The first received only DP milk instead of STD, the second received commercial UHT 1.5% cow milk instead of STD, both equilibrated at a daily dose to the chow protein level (4 g of protein per rat per day, equal to 135 mL of milk per rat daily), while the third group served as a control, receiving only STD. The rats were monitored for 15 days. All groups had water *ad libitum*. The body weights of control and milk fed rats were measured before and at the end of the experiment. The animals were killed after ketamine anesthesia injected intramuscularly at a dose of 90 mg/kg body weight. When anesthesia was achieved, blood was drawn from the abdominal aorta after opening the peritoneal cavity. When animals were sacrificed by exsanguination, the organs were harvested for further analysis. All animals were consecutively sacrificed in the same way from each group. The thymus was isolated from surrounding connective tissue and trachea using sterile technique. The obtained thymuses were placed in phosphate-buffered saline (PBS), dried on filter paper and measured. The spleens were extirpated, washed in ice-cold PBS and immediately placed in an ice bath. All other organs (heart, kidneys and liver) were immediately washed in ice-cold PBS and frozen for further analyses. At the end of the experimental protocol, the animals were sacrificed by cervical dislocation. Animal corpses, considered as biological waste, were forwarded to the Veterinary Institute of Nis (Serbia) for safe disposal.

### Bone marrow, thymocyte and spleen isolation

For each rat included in the experiment, the head of a single femur was cut off and bone marrow was removed by aspirating PBS through a puncture in the other end of the bone using a needle and a syringe. The marrow recovered from the femur was suspended in 1 mL of PBS. The obtained bone marrow suspensions were placed in an 80-mesh stainless steel sieve on top of a plastic tube. The samples were kept at −20 °C before analysis. Thymocytes were released by teasing each thymus through the steel mesh, as described in our previous report^61^. Tissue homogenate samples of thymocytes and bone marrow were obtained by using frozen tissues which were homogenized with ice using laboratory homogenizer.

### Determination of MyD88, Akt-1 kinase/phospho-Akt-1 kinase, p38/phospho-p38, CD4+ and CD8+ antigens in rat thymocytes and bone marrow

Immunofluorescence technique using primary, unlabeled antibody and fluorophore-labeled (fluorescein isothiocyanate, FITC) secondary antibody was standardized in our laboratory, as explained in detail in our previous articles^6^,^7^,^61^. All antibodies were purchased from Santa Cruz Biotechnology (USA): MyD88 (B-1 sc-136970), Akt-1 (5c10: sc-81434), phospho-Akt-1 (11E6: sc-81433 mouse monoclonal IgG_1_), NF-κB (p65 C-20: sc-372 epitope mapping at the C-terminus of NF-κB p65), p38 (p38α/β A-12: sc-7972), phospho-p38 (epitope corresponding to phosphorylated Tyr 182), CD4+ (H-370, sc-7219) and CD8+ (H-160 sc-7188). Briefly, the procedure was performed in the following steps: (i) *protein immobilization* on a solid polystyrene microtiter plate by pipetting of 10 μL of each protein sample in flat-bottomed, 96-well plates, two plates for each antigen (serving as the test and the control plates). The antigens were allowed to attach at 4 °C overnight using a carbonate/bicarbonate coating buffer (100 mM, pH 9.6); (ii) *antigen binding* via the incubation of test plates with the corresponding primary antibody at 4 °C for 24 hours; (iii) *detection assay with secondary antibody* in which each antibody test and control plates were washed and incubated with the corresponding FITC-conjugated antibody (sc-7972 FITC) for 2 hours in the dark. The excess secondary antibodies were washed; (iv) *fluorescence detection,* analyzed on a Victor Perkin Elmer-Wallac multiplate reader. The fluorescence of the corresponding control plates in which the primary antibody was omitted was subtracted from the corresponding test plate values, and the quantity was expressed as the logarithm of fluorescence per quantity of cellular proteins.

### Determination of tissue DNase

The activity of acid and alkaline DNase was measured in tissue homogenate samples spectrophotometrically at 260 nm, according to the method of Bartholeyns *et al*.^62^. The method is based on acid-soluble nucleotide determination after precipitation using specific buffers and activators. Enzyme activity was expressed as U/g protein according to the corresponding standards, commercial DNase I and DNase II, purchased from Sigma-Aldrich (USA).

### Caspase-1 determination

Caspase-1 activity was measured using a caspase assay kit from RD Diagnostics (Greece). The activity of caspase-1 from the in tissue homogenate samples was measured using the caspase-1 colorimetric substrate WEHD-p-nitroaniline (pNA). The plates were read at 405 nm. The activity was expressed as U/g protein.

The amount of total protein content of in tissue homogenate samples of bone marrow and isolated thymocytes was quantified by the method described by Lowry *et al*.^63^.

### *In vitro* apoptosis assay of isolated thymocytes

Thymocytes were prepared from the thymuses of Wistar rats, as described. The cells were seeded in 96-well plates (Sarstedt, Nümbrecht, Germany) (1 × 10^6^ cells/well) and cultivated with UHT milk, DP milk (both at 1:100 and 1:50 dilutions) or with control medium (RPMI 1640 medium containing 10% fetal calf serum) for 20 hours. The positive control was dexamethasone (100 ng/mL). After cultivation, apoptosis was assessed using the Dead Cell Apoptosis Kit with Annexin V FITC and propidium iodide (PI) (Invitrogen, Carlsbad, CA, USA), assessed by flow cytometry, according to the manufacturer’s instructions. This assay enabled the identification of early apoptotic (Annexin-V-FITC+/PI−) cells, late apoptotic/secondary necrotic (Annexin-V-FITC+/PI+) cells, and primary necrotic (Annexin-V-FITC−/PI+) cells.

### Blood collection, hematological parameters and cytokine analysis

Obtained blood was transferred to an Eppendorf tube containing 50 μL 0.01 M EDTA. The hematocrit (Hct), total erythrocyte (RBC) count, mean corpuscular volume of erythrocytes (MCV), mean content of hemoglobin in single erythrocytes (MCH), mean concentration of hemoglobin in erythrocytes (MCHC), distribution of red anisocytosis width (RDW), hemoglobin (Hb) concentration, leukocyte count (WBC) and leukocyte formula (percent of lymphocytes, monocytes and neutrophils), platelet count, plateletcrit (PCT), mean platelet volume (MPW) and the width of the distribution of platelets (PDW) were analyzed in the obtained blood. All analyses were performed using a Dade Behring RxL Max automatic analyzer. Cytokines TGF-β and IL-6 were measured by using standard Quantikine ELISA test (Minneapolis, MN, USA) for quantitative determination of activated TGF-β and MyBioSource ELISA test (San Diego, CA, USA), respectively. The level was expressed as percent of control value.

### Streptozotocin-diabetes model

Male Sprague-Dawley rats weighting 200 ± 10 g were used. The rats were allocated into eight experimental groups, the experiment lasted 14 days. Streptozotocin diabetes was induced by a single intraperitoneal injection of Streptozotocin (Sigma, St. Louis USA) in a dose of 62 mg/kg body weight, according to the defined protocol^64^. Control rats received an equal volume of solvent. The following eight groups were allocated: (i) control group receiving standard diet (STD); (ii) group fed DP milk for 14 days, instead of STD, according to the previous protocol; (iii) group fed commercial UHT 1.5% cow milk instead of STD, for 14 days, according to the previous protocol; (iv) group fed DP milk for 7 days before the streptozotocin injection and continued for next 7 days after the streptozotocin injection; (v) group fed commercial UHT 1.5% cow milk for 7 days before the streptozotocin injection and continued for next 7 days after the streptozotocin injection; (vi) group received simultaneously streptozotocin injection and fed DP for next 7 days; (vii) group received simultaneously streptozotocin injection and fed commercial UHT 1.5% cow milk for next 7 days; (viii) group received streptozotocin injection and fed STD for next 7 days. No insulin was administered. All experimental groups were given water *ad libitum*. The animals were then housed in a 12 hours light/dark cycle. Animals were sacrificed after 7 days of streptozotocin injection. The diabetic state was confirmed by the measuring of blood glucose levels.

### Statistics

The ANOVA test for multivariate analysis was carried out to evaluate the statistical significance between groups. Significance levels were expressed as *P < 0.05; **P < 0.01; ***P < 0.001; and not significant (NS).

## Additional Information

**How to cite this article**: Kocic, G. *et al*. Depurinized milk downregulates rat thymus MyD88/Akt/p38 function, NF-κB-mediated inflammation, caspase-1 activity but not the endonuclease pathway: *in vitro*/*in vivo* study. *Sci. Rep.* **7**, 41971; doi: 10.1038/srep41971 (2017).

**Publisher's note:** Springer Nature remains neutral with regard to jurisdictional claims in published maps and institutional affiliations.

## Figures and Tables

**Table 1:** Blood cell characteristics, plasma protein concentration, body weight (BW) and spleen wet weight in rats fed different milk diets and control rats.

Blood parameters	DP milk fed group (8 rats)	UHT commercial milk fed group (8 rats)	Control group (8 rats)
White blood cells (WBC)			
Total WBC count (×10^9^/L)	3.51 ± 1.62^•^	4.70 ± 1.77*	3.15 ± 1.45
Ly (%)	71.60 ± 5.67^•^	66.70 ± 4.31*	74.40 ± 5.54
Mo (%)	13.66 ± 7.65*^•^	18.03 ± 3.38	20.37 ± 4.83
Ne (%)	15.45 ± 3.53***	15.20 ± 7.65***	5.22 ± 4.07
Red blood cells (RBC) and hematocrit (Hct)			
Total RBC count (×10^12^/L)	6.85 ± 0.96	6.44 ± 0.45*	6.95 ± 0.26
MCV (μm^3^)	54.8 ± 0.94	55.00 ± 1.71	56.48 ± 1.46
Hemoglobin, Hb (g/L)	145.16 ± 15.43^•^	134.33 + 8.07*	148.00 ± 5.60
MCH (pg)	21.30 ± 0.93	20.83 ± 0.57	21.20 ± 0.50
MCHC (g/L)	388.33 ± 11.9^•^	379.00 ± 9.67	376.57 ± 4.46
RDW (%)	14.21 ± 1.01	13.58 ± 0.72*	14.44 ± 0.49
Hct (%)	37.50 ± 4.95	35.46 ± 2.95*	39.28 ± 1.27
Spleen wet weight (wW) and Body weight			
Spleen wW(g)	0.281 ± 0.034***^•^	0.228 ± 0.029***	0.405 ± 0.040
BW (start/end)(g)	207 ± 9.05/198 ± 12.32	199 ± 10.05/188 ± 10.24	210 ± 10.34/204 ± 9.65
Platelets (PLT)			
PLT (×10^9^/L)	735.5 ± 150.92*^•^	881.83 ± 180.96	954.00 ± 101.83
PCT (%)	0.17 ± 0.047**^•^	0.21 ± 0.057	0.23 ± 0.075
MPV (fL)	2.43 ± 0.31*	2.48 ± 0.30*	3.05 ± 0.64
PDW (%)	16.45 ± 1.09	16.85 ± 0.70	16.50 ± 1.23
Plasma proteins			
Total proteins (g/L)	56.18 ± 2.65***^••^	59.28 ± 5.69	60.53 ± 2.65
Albumin (g/L)	29.33 ± 1.97***	31.00 ± 4.69***	37.66 ± 7.47

***P < 0.001 **P < 0.01 *P < 0.05 vs control. ^•••^P < 0.001 ^••^P < 0.01 ^•^P < 0.05 DP vs UHT. White blood cell count (WBC), leukocyte formula (percent of lymphocytes, monocytes and neutrophils), red blood cell (RBC) count, mean corpuscular erythrocyte volume (MCV), mean hemoglobin content in single erythrocyte (MCH), mean erythrocyte hemoglobin concentration (MCHC), RBC distribution width (RDW), hemoglobin concentration (Hb), hematocrite percent (Hct), platelet count (PLT), plateletcrit (PCT), mean platelet volume (MPV), platelet distribution width (PDW), total plasma proteins and plasma albumin concentration were analyzed by using Dade Behring RxL Max automatic analyzer.
